# Prediction of dysphagia aspiration through machine learning-based analysis of patients’ postprandial voices

**DOI:** 10.1186/s12984-024-01329-6

**Published:** 2024-03-30

**Authors:** Jung-Min Kim, Min-Seop Kim, Sun-Young Choi, Ju Seok Ryu

**Affiliations:** 1 Department of Health Science and Technology, Graduate School of Convergence Science and Technology, Seoul National University, Seoul, South Korea; 2 Department of Rehabilitation Medicine, Seoul National University Bundang Hospital, Seongnam, South Korea; 3 Department of Multimedia Engineering, Dongguk University, Seoul, South Korea; 4 Seoul National University College of Medicine, 82 Gumi-Ro 173 Beon-Gil, Bundang-Gu, Seongnam-Si, Seoul, Gyeonggi-Do 13620 South Korea

**Keywords:** Dysphagia aspiration, Postprandial voice-based, Disease prediction model, Machine learning, Remote diagnosis and monitoring technology, Voice analysis

## Abstract

**Background:**

Conventional diagnostic methods for dysphagia have limitations such as long wait times, radiation risks, and restricted evaluation. Therefore, voice-based diagnostic and monitoring technologies are required to overcome these limitations. Based on our hypothesis regarding the impact of weakened muscle strength and the presence of aspiration on vocal characteristics, this single-center, prospective study aimed to develop a machine-learning algorithm for predicting dysphagia status (normal, and aspiration) by analyzing postprandial voice limiting intake to 3 cc.

**Methods:**

Conducted from September 2021 to February 2023 at Seoul National University Bundang Hospital, this single center, prospective cohort study included 198 participants aged 40 or older, with 128 without suspected dysphagia and 70 with dysphagia-aspiration. Voice data from participants were collected and used to develop dysphagia prediction models using the Multi-Layer Perceptron (MLP) with MobileNet V3. Male-only, female-only, and combined models were constructed using 10-fold cross-validation. Through the inference process, we established a model capable of probabilistically categorizing a new patient's voice as either normal or indicating the possibility of aspiration.

**Results:**

The pre-trained models (mn40_as and mn30_as) exhibited superior performance compared to the non-pre-trained models (mn4.0 and mn3.0). Overall, the best-performing model, mn30_as, which is a pre-trained model, demonstrated an average AUC across 10 folds as follows: combined model 0.8361 (95% CI 0.7667–0.9056; max 0.9541), male model 0.8010 (95% CI 0.6589–0.9432; max 1.000), and female model 0.7572 (95% CI 0.6578–0.8567; max 0.9779). However, for the female model, a slightly higher result was observed with the mn4.0, which scored 0.7679 (95% CI 0.6426–0.8931; max 0.9722). Additionally, the other models (pre-trained; mn40_as, non-pre-trained; mn4.0 and mn3.0) also achieved performance above 0.7 in most cases, and the highest fold-level performance for most models was approximately around 0.9. The ‘mn’ in model names refers to MobileNet and the following number indicates the ‘width_mult’ parameter.

**Conclusions:**

In this study, we used mel-spectrogram analysis and a MobileNetV3 model for predicting dysphagia aspiration. Our research highlights voice analysis potential in dysphagia screening, diagnosis, and monitoring, aiming for non-invasive safer, and more effective interventions.

*Trial registration:* This study was approved by the IRB (No. B-2109-707-303) and registered on clinicaltrials.gov (ID: NCT05149976).

**Supplementary Information:**

The online version contains supplementary material available at 10.1186/s12984-024-01329-6.

## Introduction

Dysphagia is a difficulty in swallowing food normally due to impaired movement in swallowing-related organs, which increases the risk of food passing into the airway [1]. The most common diagnostic method, the Videofluoroscopic Swallowing Study (VFSS), requires specialized equipment typically found only in hospitals, resulting in long wait times and radiation risks [2–4]. In addition to the VFSS, various other diagnostic methods for dysphagia, such as Fiberoptic Endoscopic Evaluation of Swallowing (FEES), manomety, and laryngeal electromyography. However, each of these methods has its own limitations. [5–9] For example, FEES can only evaluate the pharyngeal stage and carries the risk of complications such as anterior or posterior epistaxis, and laryngospasm. [6] Meanwhile, manometry requires invasive procedures, and both manometry and laryngeal electromyography remain challenging to analyze [7–9]. Thus, the current dysphagia diagnostic methods in clinical settings are limited in their ability to continuously monitor changes in a patient's condition over time [10].

## The state of the art

To overcome the limitations of existing diagnostic test methods for dysphagia conducted in hospitals, such as VFSS, researches have focused on non-invasive testing methods for dysphagia, particularly aspiration, in various previous studies. The 3-oz water swallow test showed a sensitivity of 59–96.5% and specificity of 15–59% when compared with VFSS and FEES [11–13]. The Gugging swallowing screen test had a sensitivity of 100% and a specificity of 50–69% in acute stroke patients [14]. Sensitivity and specificity for dysphagia based on language and speech-related dysfunctions were reported as follows: aphasia (36% and 83%, respectively), dysarthria (56% and 100%, respectively), and a combination of variables (64% and 83%, respectively) [15]. Dysphonia, dysarthria, gag reflex, cough, and voice changes were used as diagnostic performance measures [16]. Other screening tools, such as the Functional Oral Intake Scale (FOIS), modified Mann assessment of swallowing ability test, and volume-viscosity swallow test (V-VST), etc., were also developed and subjected to performance validation [13, 17–25].

While predictive performance varies depending on the research techniques, all of them require expert intervention for accurate diagnosis and monitoring, which limits their applicability for everyday life monitoring. Therefore, recent research endeavors to develop technologies for diagnosing and monitoring patients with dysphagia using their voices, driving researchers to explore novel approaches in clinical settings [26–31]. The efforts to utilize patients' voices in diagnosing dysphagia were influenced by alterations in airway vibrations caused by food aspiration, resulting in changes in voice quality and parameters [24, 31, 32]. Most previous studies on voice analysis in patients with dysphagia have focused on analyzing frequency perturbation measures (Relative Average Perturbation (RAP), Jitter percent, Pitch Period Quotient (PPQ), etc.), amplitude perturbation measures (Shimmer Percent (SHIM), Amplitude Perturbation Quotient (APQ), etc.), and noise analysis (Noise to Harmonic Ratio (NHR)) to differentiate between high- and low-risk groups due to aspiration into the airway [26–31]. Additionally, vocal intensity (Maximal Voice Intensity (MVI)) and vocal duration measures (Maximum Phonation Time (MPT)) were used as voice analysis indicators [26]. Moreover, some studies have analyzed the correlations between these measures and established clinical diagnostic indicators for dysphagia, such as the Penetration-Aspiration Scale (PAS), Videofluoroscopic Dysphagia Scale (VDS), and American speech-language-hearing association national outcome measurement system swallowing scale (ASHA-NOMS) [26]. Some studies have employed the Praat program to extract these sound parameters and analyze each indicator, either using voice-only or combining voice with clinical data indicators, trained with algorithms such as Logistic Regression, Decision Tree, Random Forest, Support Vector Machine (SVM), Gaussian Mixture Model (GMM), and XGBoost [27]. Another study reported the results of dysphagia prediction using specific phonation or articulation features trained using SVM, random forest, and other methods [28]. However, these studies often extracted specific vocal numerical parameters rather than analyzing the patient's voice itself, which may limit their universal application in diagnosis and monitoring.

We hypothesized that patients with dysphagia may experience changes in their voice due to weakened muscles and aspiration below the vocal folds. Additionally, it is assumed that a more precise assessment can be achieved through the application of machine learning to analyze patients' voices. Based on this hypothesis, the primary objective of this study was to explore the efficacy of machine learning into predicting dysphagia by analyzing the post-prandial voices of patients. In this study, we developed a dysphagia prediction model using the entire voices of patients, represented as mel-spectrograms. Furthermore, we applied the Efficient Pre-trained CNNs for Audio Pattern Recognition (EfficientAT model, MIT license) algorithm, developed for audio classification problems, to our dysphagia data [33, 34]. The significance of this study is highlighted by our analysis of the entire voice of patients using mel-spectrograms and applying the EfficientAT model for the first time in a clinical setting for patients with dysphagia. The ultimate goal was to establish the groundwork for the future development of an advanced dysphagia diagnosis and monitoring system.

## Methods

### Study design

This single-center, prospective study was conducted from October 2021 to February 2023 at the Seoul National University Bundang Hospital. The study protocol was approved by the Seoul National University Bundang Hospital Institutional Review Board (IRB No.: B-2109-707-303, First approval date: 2021.09.01, Approval expiration date: 2024.08.31, Actual study start date: 2021.10.07, Actual study completion date: 2023.02.28, Research type: Investigator Initiated Trial (IIT)) and registered at clinicaltrials.gov (ClnicalTrial.gov ID: NCT05149976, Initial release: 2021.11.01, Last release: 2023.05.09). Participants with dysphagia symptoms underwent Videofluoroscopic Swallowing Study (VFSS), and with their guardians, received research information and consent forms from occupational therapists. Considering future applications in medical device development and the difficulties in recruiting normal participants in the hospital, additional healthy volunteers were recruited through notices on in-house bulletin boards and online announcements. The skilled occupational therapist and clinical dietitian provided detailed study explanations before obtaining informed consent. The two clinicians made the final determination of eligibility for study participation based on a comprehensive review, considering factors like age, gender, underlying conditions, signs of dysphagia, and VFSS results. This study was conducted in accordance with the strengthening the reporting of observational studies in epidemiology (STROBE) guidelines.

### Participants

The inclusion criteria for selecting study subjects are as follows: patients (1) who have signs and symptoms of dysphagia and are scheduled for VFSS, (2) can record ‘Ah ~ for 5 s’, and (3) healthy volunteers without dysphagia symptoms who can record voice as a normal. The exclusion criteria were as follows: (1) inability to speak according to the researcher’s instructions, (2) patients whose VFSS was reexamined, and (3) serious voice disorders (such as vocal nodules, vocal fold paralysis, vocal fold muscle tension dysphonia, etc.).

**Fig. 1:**
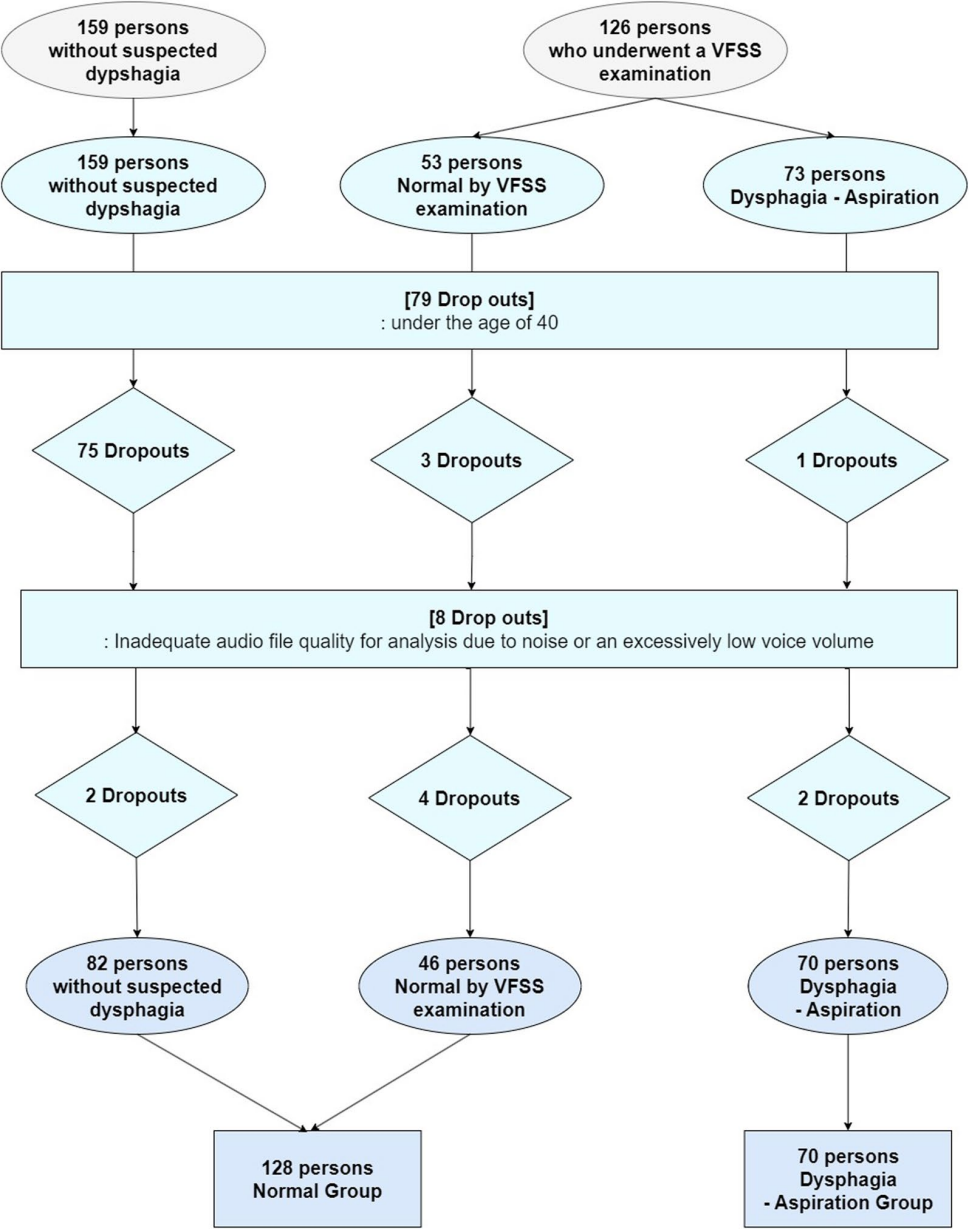
Flowchart of the dysphagia voice cohort

The determination of normal in healthy volunteers was made through telephone interview surveys that recorded the presence or absence of dysphagia symptoms, as well as age, gender, and comorbid conditions. Among those assessed with VFSS, normal or the presence of aspiration was classified based on the results of the VFSS: individuals with the Penetration-Aspiration Scale (PAS) 1 were considered normal, while those with the PAS 5–7 were classified as aspiration. The results for 126 participants (53 normal, 73 aspiration) who underwent VFSS were assessed based on images, interpreted by two clinical physicians. A reliability test yielded a Cohen's Kappa coefficient of 0.87. The final determination of the degree of dysphagia was made by consensus between two clinicians. Voice recordings were obtained with the consent of 285 participants, including 159 individuals without suspected dysphagia (healthy volunteers) and 126 who underwent VFSS because of suspected dysphagia aspiration. In the patient group, 1 participant aged < 40 years was included in the aspiration subgroup. To eliminate age-related bias in the patient's voice-based predictive model, 79 participants under the age of 40 years (comprising 75 participants without suspected dysphagia, 3 participants from the normal group by VFSS examination, and 1 participant from the aspiration group) were excluded from the study population. 8 participants (2 participants without suspected dysphagia, 4 participants from the normal group by VFSS examination, and 2 participant from the aspiration group) with poor audio quality were excluded from the collected recordings. The final study population consisted of 198 participants, categorized into the normal group (128 participants, including both individuals without suspected dysphagia and those who received a normal diagnosis based on VFSS), and the aspiration group (70 participants), based on VFSS interpretations by physicians. Figure 1 shows detailed flow chart of the recruitment of research subjects.

### Voice recording procedures

After obtaining consent from the patient, a VFSS was performed using the modified Logemann protocol which is commonly used in domestic hospitals, to evaluate dysphagia. [35] During the test, the patient was instructed to repeat the sound 'Ah ~ ' once or more for at least 5 s after consuming water, fluid thickening with level 3 (FT3), liquid food (LF), semi-blended diet (SBD), small fluid (SF), and yoplait (YP), while their voice was recorded using a Sony ICD-TX660 recorder (bit resolution: 16 bit, bit rate range: 32–192 kbps, actual recorded bit rate: 64 kbps, sampling frequency: 44.1 kHz, microphone bandwidth: 95–20,000 Hz, recording: stereo) while limiting intake to 3 cc. Researchers instructed the participants from outside the soundproof window in the VFSS examination room, ensuring an environment isolated from external noise. For the healthy volunteers, which consisted of subjects without dysphagia, their voices were recorded in a separate, noise-reduced room under the guidance of the researchers, and once or more for at least 5 s after drinking water using a voice recording function on a mobile device. The recording was conducted with the recording device placed on the upper sleeve of the patient's clothing.

The similarity between devices was assessed by preprocessing voice data as outlined in the Voice Data Preprocessing section, and then converting it into Mel spectrograms to test using cosine similarity. This method measures the similarity between two datasets by utilizing the cosine angle between two vectors. Additional file 1: Table S1 highlights the negligible impact of devices and positions, adhering to the study’s protocol. The Sony recorder was exclusively used at the upper sleeve position, whereas the mobile phones (Samsung and iPhone) were assessed at three distinct locations: the examiner’s upper sleeve, on a table, and in front of the mouth. This method enabled a direct comparison of data from mobile phones at each location with the Sony recorder’s sleeve data and assessed the cosine similarity between the mobile phones across the three positions. Additional file 2: Table S2 further investigates the effect of position within the same device, presenting results from recordings at the three positions and specifically focusing on the positional impact within each device. This comprehensive analysis determined that device type, and position have a minimal effect on audio quality. All devices, including Samsung phones, iPhones, and the Sony recorder, showed similarity scores above 0.8, indicating no significant variance between devices or positions when subjected to the same preprocessing techniques. The testing was conducted by recording simultaneously with the same protocol and subject using three different recorders and then assessing the similarity of the recorded data.

In total, 403 voice files were collected, consisting of 210 files from the normal group (64 files for men, 146 files for women) and 193 files from the aspiration group (147 files for men, 46 files for women).

### Voice data preprocessing

**Fig. 2:**
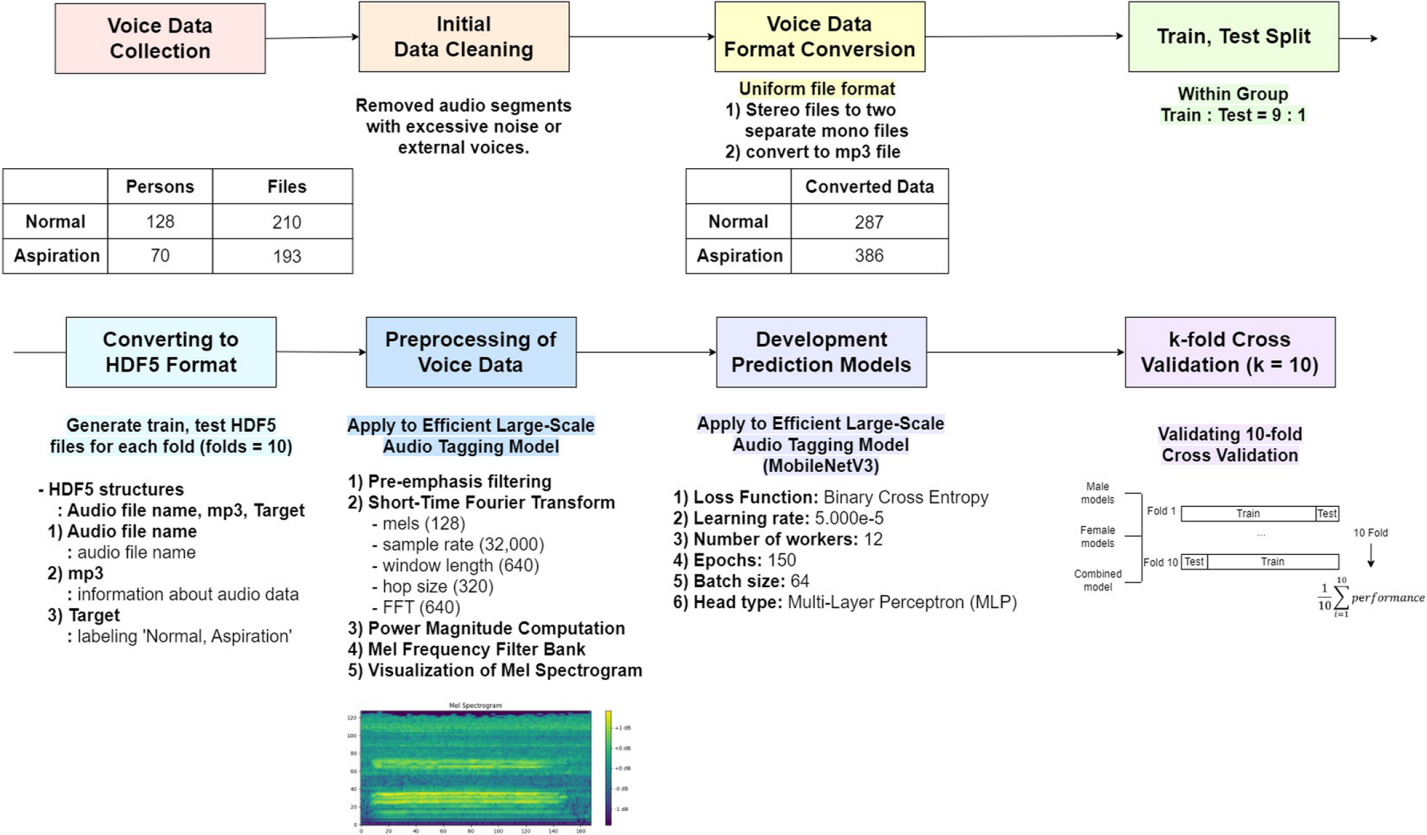
Overview of voice data preprocessing and modeling. The voice data collected from normal and aspiration subjects is preprocessed through the following steps, then used to create a prediction model through training, and subsequently evaluated using 10-fold Cross-Validation 1) Initial Data Cleaning in Voice Recording: To reduce background noise and external voices, researchers individually reviewed all audio data and removed segments with excessive noise or external voices 2) Voice Data Format Conversion: To standardize audio files for machine learning and reduce bias from recording environments, we converted stereo files to two separate mono files and standardized all audio formats to mp3 at 64kbps 3) Train, Test split: The mp3-formatted data were split into training and testing sets in a 9:1 ratio and then divided into ten subsets for 10-fold cross-validation, ensuring data from the same individual remained in the same fold 4) Converting to HDF5 Format: We converted voice data into HDF5 format, structuring the data information with audio file names, audio (mp3) information, and numerical labels for normal or aspiration 5) Preprocessing of Voice Data: Voice preprocessing was conducted using the EfficientAT model, involving transformation into Mel spectrograms with specific hyperparameters and techniques like STFT 6) Development Prediction Models and 10-fold Cross-Validation: MobileNet V3 was used for voice training with binary cross-entropy loss, comparing pre-trained (mn30_as, mn40_as) and non-pre-trained (mn3.0, mn4.0) models and predictive performance, validated using 10-fold cross-validation and trained with specific settings including MLP head type

Following the procedure outlined in Fig. 2, preprocessing was conducted on the voice data, and based on this, a machine learning model was constructed.

*Step 1. Initial data cleaning in voice recording*

To minimize background noise and external voices, all audio data was initially reviewed individually by researchers, and segments with excessive noise or external voices were uniformly trimmed.

*Step 2. Conversion of voice data format*

To make audio files suitable for machine learning and minimize bias due to recording environments, we performed two steps: (1) Files recorded in stereo (due to the characteristics of the Sony recorder) were all converted to mono. To minimize data loss, each side’s data (right and left) was split to form two mono files. Files originally recorded in mono were used as is. (2) For voice data standardization, audio files in various formats like wav, m4a, and mp3 were all converted to a unified format of mp3 at 64kbps. As a result, 673 data files (287 normal group files: men (94 files), women (193 files), 386 aspiration group files: men (294 files), women (92 files) were converted to mp3 format and utilized for model development. We analyzed the degree of data loss resulting from the compression of original audio files into 64kbps mp3 format across various file extensions. This analysis, detailed in Additional file 3: Table S3, utilized Mean Squared Error (MSE) and Peak Signal-to-Noise Ratio (PSNR) to evaluate the loss. The process confirmed the differences between the original audio data and the mp3 64kbps converted data, directly from the waveform of the source audio itself, prior to the preprocessing described in Step 5. The average MSE for the entire audio source was calculated to be Mean ± SD (0.0002 ± 0.0002), and the PSNR was Mean ± SD (35.95 ± 3.76). Given that a PSNR between 30 to 40 dB is indicative of good quality preservation, according to the literature, and quality should be maintained without a degradation exceeding 10 to 20 information bits, this suggests that the conversion process to mp3 at 64kbps effectively preserves good quality without resulting in significant loss. [36]

*Step 3. Creation of train and test dataset for k-fold cross validation*

The mp3-formatted data were divided into training and testing sets in a ratio of approximately 9:1 for each group. For 10-fold cross-validation, the data has been divided into ten subsets based on individuals in each group. In other words, data from the same person is grouped together in the same fold. The range of these sections was varied to create 10-fold cross-validation datasets.

*Step 4. Conversion of voice data to hdf5 format for model training*

To train MobileNet V3 with an Efficient Pre-trained CNNs for Audio Pattern Recognition (EfficientAT model, MIT license), we converted the data into a suitable format. This was achieved by modifying the create_h5pymp3_dataset.py code from PaSST: Efficient Training of Audio Transformers with Patchout (PaSST, Apache-2.0 license) research and transforming the training/test data into HDF5 format files [37, 38]. The structure of the transformed HDF5 data consisted of the audio file name, audio data information in mp3 format, and labeled information on normal, or aspiration in numeric form.

*Step 5. Preprocessing of voice data*

Voice preprocessing was conducted using an EfficientAT model, which is widely utilized for audio classification tasks [33, 34] This process involved defining the ‘MelSTFT’ class for converting audio waveforms into Mel spectrogram format suitable for machine learning. It consists of several steps, including pre-emphasis filtering, short-time Fourier transform (STFT), power magnitude computation, and a Mel frequency filter bank. The hyperparameters, such as the number of mels (128), sample rate (32,000), window length (640), hop size (320), and the number of Fast Fourier Transforms (FFT, 640), control the preprocessing process. With the given hyperparameters, the time shift (hop size) is 10 ms, and the window length is 20 ms. The parameters used for analysis were set considering the available GPU capacity, the recorder’s LPCM (Linear Pulse Code Modulation, 44.1 kHz), performance, and the storage capacity of the final model. In summary, this process enables to transform audio data into a perceptually related Mel-spectrogram representation.

### Development of dysphagia prediction models

MobileNet V3 was utilized as the machine learning technique for voice training. Binary cross entropy with logits loss was used as the loss function to evaluate the predictive performance of the algorithm [33, 34]. The two pre-trained models were named mn30_as, and mn40_as in accordance with the width_mult and hyperparameters in the EfficientAT model. Similarly, two non-pre-trained models were designed with the same width_mult and hyperparameters as the pre-trained models, and were uniformly named mn3.0, and mn4.0, respectively. In the naming of models, 'mn' stands for MobileNet, a type of neural network architecture designed for use in mobile and embedded applications. The number that follows 'mn' represents the 'width_mult' parameter. For example, 'mn40_as' and 'mn4.0' indicate that the 'width_mult' parameter is set to 4.0. This particular parameter is crucial as it adjusts the width of the network, thereby directly influencing the overall size and computational demands of the model. Maintaining consistency with the naming convention of the precedent code, we have employed this same system in the EfficientAT model. Essentially, this method of naming helps in quickly identifying the architectural features and complexity of the model [33, 34]. In situations where the dataset is limited, non-pre-trained models may encounter challenges in effectively extracting features [39, 40]. Therefore, this study conducted a comparison between the pre-trained and non-pre-trained models [33, 34]. The model constructed in this manner was validated for prediction accuracy using a k-fold cross-validation with k = 10. All the models were trained for 12 number of workers, 150 epochs, and 64 batch sizes. The learning rate, using a learning rate scheduler, was initially maintained at a constant 5.00e-5, then began to decrease from around epoch 100 to 105, ultimately reaching a final learning rate of 5.00e-7. Head type has been set to Multi-Layer Perceptron (MLP).

### Outcome variables

The primary outcome of this study is the Area Under the ROC Curve (AUC), considering the imbalanced distribution of data among groups in the medical field. The ROC curve visually shows how well the model distinguishes between actual aspiration and normal cases by plotting the true aspiration rate against the false normal rate, while the AUC, which varies between 0 and 1, measures this distinction's accuracy, with values closer to 1 signifying more accurate predictions. Additionally, the degree of prediction for the model was analyzed from the perspectives of accuracy, mean average precision (mAP), sensitivity, specificity, precision, F1-score, loss, train accuracy, and train loss, and a final model was established. Accuracy is how often the model is right, the ratio of accurate predictions out of all predictions made. The mAP averages out the precision (the proportion of true positive predictions out of all positive predictions) for each class (like normal or aspiration) to get an overall score. Sensitivity checks how many of the actual aspiration cases the model correctly identified out of all the possible aspiration cases. Specificity measures how many of the actual normal cases the model correctly identified out of all the possible normal cases. Precision shows how many of the model's predicted aspiration are actually aspiration. The F1 score is a balanced average of precision and sensitivity. The loss is calculated using Binary Cross Entropy with Logits that quantifies the discrepancy between the model's predicted probabilities and the actual values for binary classifications. Train accuracy and train loss pertain to training datasets, while all other parameters are designated for assessing test datasets.

### Statistical analysis

In the Demographic characteristics section, we analyzed the distribution of gender and age in each group, which could influence individual voice characteristics, before training on the voice data. We also presented six categories of comorbid conditions that may accompany dysphagia, based on previous studies [41–43]. The distributions of gender and comorbid conditions were presented as categorical variables using Number (%) and tested using the chi-square test. Age, a continuous variable, was analyzed using Mean ± Standard Deviation (SD) and tested with the non-parametric Mann–Whitney U test due to violations of normality and sphericity, as indicated by the Shapiro–Wilk and Mauchly’s tests, respectively. The significance level for these variables was set at p < 0.05, reflecting the conventional balance between the risks of Type I and Type II errors. Model performance was primarily measured using the AUC, along with other metrics including accuracy, mAP, sensitivity, specificity, precision, F1-score, loss, train accuracy, and train loss, to provide a comprehensive view of the model's predictive performance. To enhance the model's validity, given the variability in human voices, each performance metric was calculated for each fold and then presented as an average, with a 95% confidence interval and maximum performance across 10 folds. All the analyses were conducted using Python and Google Colaboratory Pro + GPU A100. Statistical analysis and machine learning modeling were conducted between January and December 2023.

## Results

### Demographic characteristics

Table 1 shows the demographic characteristics of all the study subjects.

**Table 1:** Demographic characteristics

	Normal	Aspiration	p-value
Gender (N (%))			
Men	41 (32.03%)	52 (74.29%)	< 0.001* (χ^2^: 30.76, df: 1)
Women	87 (67.97%)	18 (25.71%)	
Age (mean ± SD)			
Total	61.16 ± 13.00	72.30 ± 12.03	< 0.001**
Men	63.27 ± 13.57	72.25 ± 11.68	0.001**
Women	60.16 ± 12.66	72.44 ± 13.34	0.001**
Comorbid conditions (N (%))			
Total			
Central nervous system disorders	17 (13.28%)	18 (25.71%)	< 0.001*** (χ^2^: 36.10, df: 5)
Digestive system and dental disorders	3 (2.34%)	12 (17.14%)	
Pulmonary disorders	4 (3.12%)	9 (12.86%)	
Other cancers	7 (5.47%)	3 (4.29%)	
Aging-related disorders	12 (9.38%)	8 (11.43%)	
None	85 (66.41%)	20 (28.57%)	
Men			
Central nervous system disorders	5 (12.20%)	11 (21.15%)	0.002*** (χ^2^: 18.54, df: 5)
Digestive system and dental disorders	1 (2.44%)	12 (23.08%)	
Pulmonary disorders	2 (4.88%)	8 (15.38%)	
Other cancers	2 (4.88%)	2 (3.85%)	
Aging-related disorders	5 (12.20%)	6 (11.54%)	
None	26 (63.41%)	13 (25.00%)	
Women			
Central nervous system disorders	12 (13.79%)	7 (38.89%)	0.140*** (χ^2^: 8.31, df: 5)
Digestive system and dental disorders	2 (2.30%)	0 (0.00%)	
Pulmonary disorders	2 (2.30%)	1 (5.56%)	
Other cancers	5 (5.75%)	1 (5.56%)	
Aging-related disorders	7 (8.05%)	2 (11.11%)	
None	59 (67.82%)	7 (38.89%)	

^*^The Chi-square test results show a significant difference. To address gender bias, separate models were constructed for each gender (male and female). The data was then divided into 10 folds for each gender. After that, the results were combined in the gender-neutral model, effectively removing any gender-related biases

^**^The Mann–Whitney U test results indicate a significant difference between the two groups. However, to eliminate bias, participants under the age of 40 were excluded from the analysis

^***^Regarding the comorbid conditions, a Chi-square test was conducted for analysis. While there are no significant differences observed among females, statistically significant differences are found in the overall dataset or males. However, vocal fold-related conditions were excluded, and dysphagia can occur in conjunction with various other conditions, which may account for differences when compared to the normal group

### Model performance

For the 10-fold cross-validation, male-only, female-only, and combined (men + women) models were constructed. Table 2 shows the average predictive performance of the combined (men + women) model across 10 folds. Regarding the primary outcome, the average AUC values were mn40_as = 0.8275 (95% CI 0.7643–0.8908; max in 10 folds 0.9500) and mn30_as = 0.8361 (95% CI 0.7667–0.9056; max in 10 folds 0.9541) for the pre-trained models and mn4.0 = 0.8039 (95% CI 0.7378–0.8700; max in 10 folds 0.9691), mn3.0 = 0.8177 (95% CI 0.7601–0.8753; max in 10 folds 0.9561) for the non-pre-trained models. Owing to the smaller amount of available data, the pre-trained models (mn40_as and mn30_as) demonstrated higher performance than the non-pre-trained models (mn4.0 and mn3.0). In addition, all models consistently showed high prediction accuracy in analyzing a person's voice, with metrics such as accuracy, mAP, sensitivity, specificity, precision, and F1-score exceeding approximately 70% or 0.7.

**Table 2:** The levels of prediction for combined (men + women) model

Model	Pre-trained models		Non-pre-trained models	
	mn40_as	mn30_as	mn4.0	mn3.0
*AUC (Area under the curve)*				
AUC average (95% CI)	0.8275 (0.7643, 0.8908)	0.8361 (0.7667, 0.9056)	0.8039 (0.7378, 0.8700)	0.8177 (0.7601, 0.8753)
AUC max in 10 folds	0.9500	0.9541	0.9691	0.9561
*Accuracy (%)*				
Accuracy average (95% CI)	71.47 (66.73, 76.21)	77.98 (70.07, 85.89)	73.43 (68.23, 78.63)	74.98 (70.18, 79.77)
Accuracy max in 10 folds	84.91	92.45	86.90	88.68
*mAP (mean average precision, %)*				
mAP average (95% CI)	83.62 (77.74, 89.51)	84.54 (78.57, 90.52)	81.05 (75.10, 87.00)	83.07 (78.13, 88.02)
mAP max in 10 folds	95.47	95.46	97.23	95.10
*Sensitivity (%)*				
Sensitivity average (95% CI)	71.47 (66.73, 76.21)	77.80 (69.87, 85.74)	73.55 (68.34, 78.77)	74.85 (70.07, 79.63)
Sensitivity max in 10 folds	84.91	92.45	86.90	88.68
*Specificity (%)*				
Specificity average (95% CI)	72.43 (67.26, 77.60)	77.52 (69.75, 85.28)	73.16 (67.67, 78.64)	74.73 (69.01, 80.45)
Specificity max in 10 folds	85.91	93.94	88.39	90.91
*Precision (%)*				
Precision average (95% CI)	71.47 (66.80, 76.15)	77.78 (70.14, 85.42)	72.90 (68.17, 77.64)	74.06 (69.08, 79.03)
Precision max in 10 folds	84.05	91.67	85.10	88.46
*F1 Score*				
F1 Score average (95% CI)	0.7173 (0.6697, 0.7648)	0.7777 (0.6994, 0.8560)	0.7350 (0.6811, 0.7889)	0.7492 (0.7004, 0.7980)
F1 Score max in 10 folds	0.8510	0.9255	0.8720	0.8885
*Loss*				
Loss average (95% CI)	0.9225 (0.6930, 1.1520)	0.8524 (0.5410, 1.1640)	1.6013 (1.0110, 2.1920)	1.3553 (0.9250, 1.7860)
Loss max in 10 folds	1.6120	1.4136	3.1602	2.3892
*Train accuracy (%)*				
Train accuracy average (95% CI)	99.97 (99.91, 100.02)	99.98 (99.95, 100.02)	99.98 (99.94, 100.02)	99.93 (99.85, 100.02)
Train accuracy max in 10 folds	100.00	100.00	100.00	100.00
*Train loss*				
Train loss average (95% CI)	0.0017 (0.0004, 0.0031)	0.0022 (0.0014, 0.0031)	0.0010 (− 0.0001, 0.0021)	0.0052 (− 0.0024, 0.0129)
Train loss max in 10 folds	0.0070	0.0045	0.0055	0.0350

^*^All metrics represent the predictive performance on the Test Data except Train accuracy, and Train loss. The results presented in this table are the average predictive performance (95% CI) across all folds of each model after performing tenfold cross-validation

**Fig. 3:**
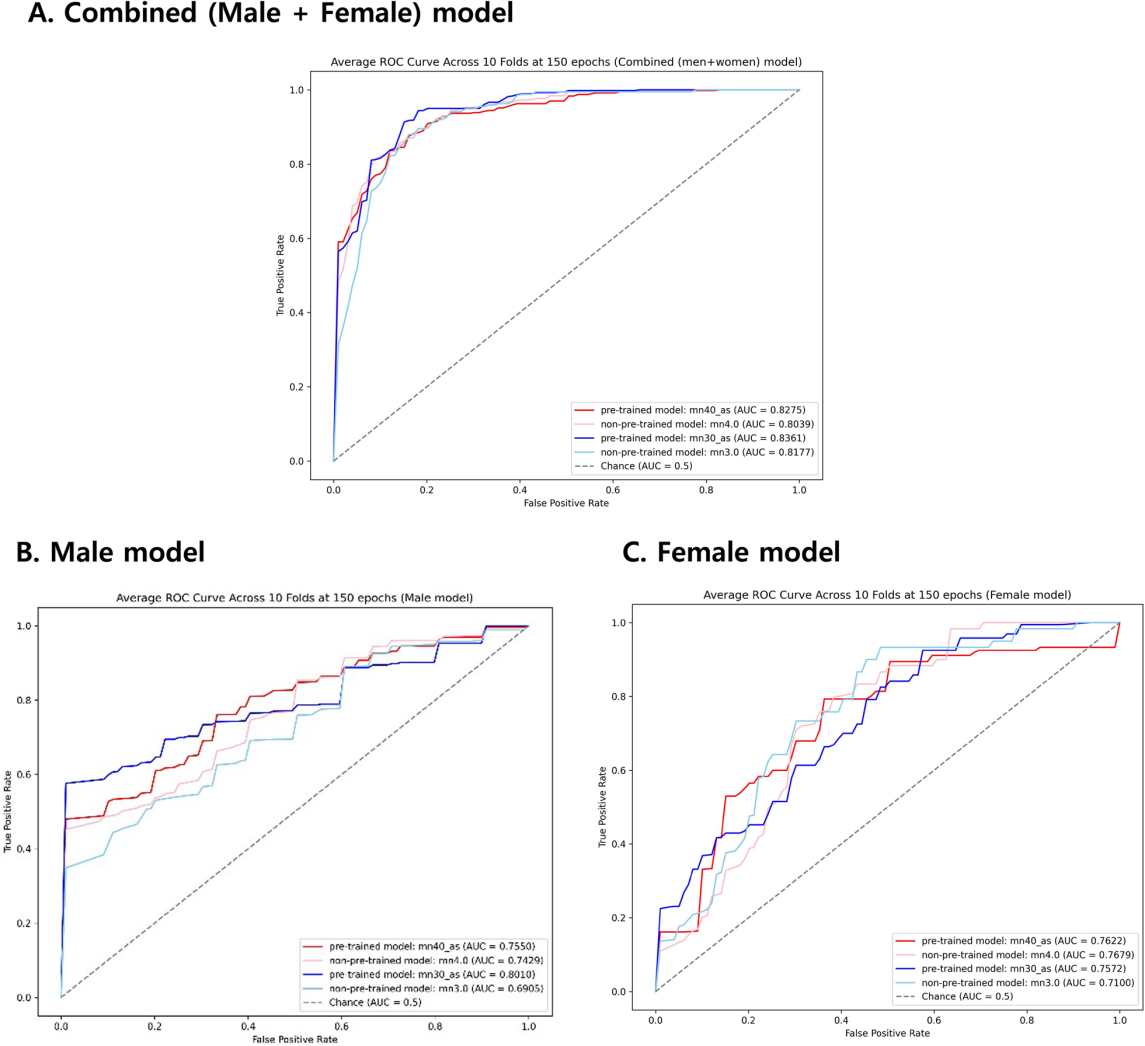
ROC curve for each prediction model. The pre-trained models demonstrated higher performance compared to the non-pre-trained models. Among the four models, the mn30_as (pre-trained model) performed the best on average in combined model and male model. However, for the female model, the mn4.0 (non-pre-trained model) was the best on average. The ROC curve was plotted, and the AUC (Area Under the Curve) was calculated

Table 3 presents the average predictive performance for each gender (men and women) across the 10 folds. The average AUC values for the pre-trained model, using mn40_as, were 0.7550 (95% CI 0.6056–0.9045; max in 10 folds 1.0000) and 0.7622 (95% CI 0.6169–0.9075; max in 10 folds 1.0000) for the male and female model, respectively. Additionally, for the pre-trained model using mn30_as, the AUC values were 0.8010 (95% CI 0.6589–0.9432; max in 10 folds 1.0000) and 0.7572 (95% CI 0.6578–0.8567; max in 10 folds 0.9779) for the male and female models, respectively. For the non-pre-trained model, using mn4.0, the AUC values were 0.7429 (95% CI 0.6262–0.8596; max in 10 folds 1.0000) and 0.7679 (95% CI 0.6426–0.8931; max in 10 folds 0.9722) for the male and female models, respectively. For the non-pre-trained model using mn3.0, the AUC values were 0.6905 (95% CI 0.5358–0.8451; max in 10 folds 1.0000) and 0.7100 (95% CI 0.5595–0.8605; max in 10 folds 0.9559) for the male and female models, respectively. Figure 3 presents the average ROC across 10 folds for each model.

**Table 3:** The levels of prediction for gender-specific model

Model	Male models				Female models			
	Pre-trained models		Non-pre-trained models		Pre-trained models		Non-pre-trained models	
	mn40_as	mn30_as	mn4.0	mn3.0	mn40_as	mn30_as	mn4.0	mn3.0
*AUC (area under the curve)*								
AUC average (95% CI)	0.7550 (0.6056, 0.9045)	0.8010 (0.6589, 0.9432)	0.7429 (0.6262, 0.8596)	0.6905 (0.5358, 0.8451)	0.7622 (0.6169, 0.9075)	0.7572 (0.6578, 0.8567)	0.7679 (0.6426, 0.8931)	0.7100 (0.5595, 0.8605)
AUC max in 10 folds	1.0000	1.0000	1.0000	1.0000	1.0000	0.9779	0.9722	0.9559
*Accuracy (%)*								
Accuracy average (95% CI)	79.44 (69.01, 89.88)	85.13 (78.07, 92.19)	78.61 (70.21, 87.01)	69.96 (58.61, 81.30)	69.17 (58.35, 79.99)	69.16 (61.76, 76.57)	69.16 (62.42, 75.89)	69.30 (61.13, 77.48)
Accuracy max in 10 folds	100.00	100.00	96.00	87.50	93.10	88.00	78.57	88.00
*mAP (mean average precision, %)*								
mAP average (95% CI)	78.13 (65.24, 91.03)	82.36 (70.38, 94.34)	76.66 (66.13, 87.19)	74.88 (62.57, 87.20)	75.69 (63.10, 88.29)	75.86 (66.33, 85.40)	74.65 (64.61, 84.69)	71.55 (59.37, 83.74)
mAP max in 10 folds	100.00	100.00	100.00	100.00	100.00	97.19	97.49	95.44
*Sensitivity (%)*								
Sensitivity average (95% CI)	79.79 (69.85, 89.73)	84.95 (77.73, 92.16)	78.61 (70.21, 87.01)	69.96 (58.61, 81.30)	69.42 (58.74, 80.10)	69.16 (61.76, 76.57)	69.16 (62.42, 75.89)	69.30 (61.13, 77.48)
Sensitivity max in 10 folds	100.00	100.00	96.00	87.50	93.10	88.00	78.57	88.00
*Specificity (%)*								
Specificity average (95% CI)	73.22 (59.93, 86.50)	75.92 (62.97, 88.86)	68.75 (57.86, 79.64)	65.39 (54.48, 76.30)	61.55 (49.89, 73.21)	64.78 (56.87, 72.70)	50.00 (50.00, 50.00)	54.65 (46.67, 62.63)
Specificity max in 10 folds	100.00	100.00	91.67	87.50	92.86	81.25	50.00	84.56
*Precision (%)*								
Precision average (95% CI)	73.57 (60.88, 86.25)	74.68 (60.26, 89.10)	71.37 (56.10, 86.63)	68.61 (57.10, 80.11)	64.87 (49.88, 79.86)	66.26 (55.97, 76.55)	34.58 (31.21, 37.94)	41.84 (28.80, 54.89)
Precision max in 10 folds	100.00	100.00	97.73	90.00	86.36	92.50	39.29	87.30
*F1 Score*								
F1 Score average (95% CI)	0.7971 (0.6997, 0.8946)	0.8317 (0.7407, 0.9228)	0.7744 (0.6855, 0.8632)	0.6973 (0.5957, 0.7989)	0.6611 (0.5449, 0.7772)	0.6878 (0.6201, 0.7555)	0.5689 (0.4829, 0.6548)	0.5962 (0.4874, 0.7051)
F1 Score max in 10 folds	1.0000	1.0000	0.9576	0.8730	0.9202	0.8710	0.6914	0.8777
*Loss*								
Loss average (95% CI)	0.8648 (0.4610, 1.2690)	0.5064 (0.2040, 0.8090)	1.1312 (0.6060, 1.6560)	1.6051 (0.8250, 2.3860)	0.9823 (0.5800, 1.3850)	1.2326 (0.4640, 2.0010)	1.0512 (0.6140, 1.4890)	0.9657 (0.5680, 1.3630)
Loss max in 10 folds	1.6027	1.1415	2.5325	4.3304	2.0750	4.0219	2.3448	1.9062
*Train accuracy (%)*								
Train accuracy average (95% CI)	99.94 (99.80, 100.08)	100.00 (100.00, 100.00)	99.97 (99.91, 100.04)	99.97 (99.90, 100.04)	100.00 (100.00, 100.00)	99.92 (99.81, 100.04)	99.92 (99.81, 100.04)	99.81 (99.61, 100.00)
Train accuracy max in 10 folds	100.00	100.00	100.00	100.00	100.00	100.00	100.00	100.00
*Train loss*								
Train loss average (95% CI)	0.0065 (− 0.0037, 0.0168)	0.0033 (0.0018, 0.0049)	0.0013 (− 0.0004, 0.0029)	0.0016 (− 0.0004, 0.0036)	0.0150 (0.0014, 0.0287)	0.0284 (0.0045, 0.0523)	0.0298 (0.0046, 0.0550)	0.0357 (− 0.0047, 0.0760)
Train loss max in 10 folds	0.0474	0.0078	0.0076	0.0092	0.0618	0.0799	0.0998	0.1849

^*^The table shows average predictive performance across all folds of each model after tenfold cross-validation

### Inference design based on trained machine learning models

**Fig. 4:**
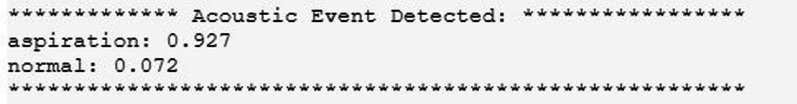
Inference. After evaluating one example of postprandial voice data that was not used during model training, it was observed that when classifying it as aspiration, the model assigned a probability of 92.7%. The output window displayed the results as mentioned earlier

The inference part is a crucial step where the trained model is applied to new patient voice data to determine if their condition is normal or dysphagia-aspiration. This process is carried out in four main stages: (1) New voice data input: We input the new patient’s voice in the same format (mp3, mono, 64kbps) used during model training. (2) Converting mp3 to waveform: The voice file is transformed into a waveform that the computer can understand and analyze. (3) Preprocessing and converting to visual representation: The voice data is processed using same settings as training (like mels (128), sample rate (32,000), window length (640), hop size (320)) to convert it into a visual format called a mel-spectrogram. (4) Loading the model and displaying results: The trained model is loaded, and it analyzes the visual representation of the voice to predict if the condition is normal or dysphagia-aspiration. The results are then displayed in a window, showing the likelihood of each condition, like Fig. 4.

## Discussion

Through this study, we developed a machine learning algorithm that can distinguish between normal and dysphagia-aspiration using postprandial voice data. This research is significant as it is the first to apply the Efficient Pre-trained CNNs for Audio Pattern Recognition, developed for existing audio classification problems (EfficientAT model, MIT license), to the differentiation of dysphagia-aspiration [33, 34]. Unlike previous studies on dysphagia patients’ voices, which primarily used voice analysis software to extract numerical data such as frequency variation, amplitude variation, and harmonics-to-noise ratio for statistical analysis or traditional machine learning methods [26–31], our study value by directly analyzing the patient’s voice itself in the form of a mel-spectrogram and learning it through deep-learning.

In the initial of research, considering that the voice patterns of men and women are different, we created separate models for males and females. Looking at the results of the tenfold cross-validation analysis for each gender, all models for male showed an average AUC (Area Under the Curve) over 0.70, with two pre-trained models showing over 0.75 (mn40_as: 0.7550, mn30_as: 0.8010). Sensitivity, an import indicator for accurately screening dysphagia aspiration patients in a clinical setting, also showed results of over 70% on average, particularly over 80% in pre-trained models (mn40_as: 79.79%, mn30_as 84.95%). For female models, the AUC indicator for all models averaged over 0.70, and similarly, both pre-trained models showed over 0.75 on average (mn40_as: 0.7622, mn30_as: 0.7572), but sensitivity was low, around 69% (mn40_as: 69.42%, mn30_as: 69.16%). This is interpreted as a limitation due to the small number of recruited aspiration female participants, totaling only 18, which was insufficient for adequate learning on actual patients. Therefore, we developed combined gender model by using both of genders’ data. As a results, the AUC for all models over 0.80, especially in the pre-trained models, which showed an average of about 0.83 (mn40_as: 0.8275, mn30_as: 0.8361), slightly higher compared to the male models. This increase is anticipated due to the increased amount of data used for learning and evaluation as both genders were studied. Regarding sensitivity, all models showed results of over 70% (particularly pre-trained model mn40_as: 71.47%, mn30_as: 77.80%), which, although relatively lower than the male models, showed higher outcomes compared to the female models. When analyzing the overall performance indicators, the pre-trained models showed higher results across all models (male, female, combined model), with the mn30_as model demonstrating the highest performance when applied to our data. This study’s results also performance comparable to previous research such as the 3-oz water swallow test and Gugging swallowing screen test, which aimed to develop non-invasive screening methods for dysphagia, thereby presenting another methods for non-invasively monitoring patients’ conditions [11–25].

In this study, the reasons for using each analytical method at each stage of the study are as follows. While there are methods such as Mel Frequency Cepstral Coefficients (MFCCs) for audio signal processing, we chose analysis through Mel-spectrograms for the following reasons: (1) Prior research indicating that the voices of patients with dysphagia are sensitive in the frequency domain was considered, emphasizing the importance of visual analysis of frequencies over time for critical signal processing and sound event characterization [27, 29–31]. (2) Transforming audio signals into Mel Spectrograms provides a perceptual and visual understanding of audio, preserving more spectral and detailed frequency information than MFCCs [44]. (3) The compatibility with CNNs (Convolutional Neural Networks) was taken into account [45, 46].

Our model also design focused on noise reduction, prediction performance, and light-weighting for mobile integration. To minimize noise, we conducted an initial data cleaning process where the patient's voice was individually reviewed, and segments with significant device noise or the presence of other voices were removed. Additionally, during the preprocessing stage using an EfficientAT model, we applied pre-emphasis filtering to reduce low-frequency components and enhance the clarity of the audio [33, 34, 47]. Both VFSS examinations and recordings of the healthy volunteers were carried out in soundproofed environments, resulting in minimal noise in the recorded voices. Regarding the second consideration, we experimented with different models including the ResNet model, which is known for its excellent performance in CNN image recognition [48, 49]. However, its accuracy was relatively low. Therefore, taking performance into consideration, we ultimately chose the current learning model. Moving on to the third consideration, we focused on model light-weighting, to achieve real-time dysphagia diagnosis, monitoring, and intervention in mobile or resource-constrained environments. We converted the audio data from stereo to mono format, improving efficiency by eliminating the need for simultaneous processing of the two channels and enhancing voice recognition accuracy [50]. Additionally, we unified and compressed the files into mp3 format for real-time processing on mobile devices and medical devices [51, 52]. Studies have reported the existence of data loss in voice due to the compressed nature of the mp3 format [53]. However, prior research related to mp3 compression has shown that for mp3s with compression rates between 56 and 320 kbps, the loss rate was less than 2% for small mean errors based on the f_0_, and less than 1% for pitch range [52]. Given the low loss rate reported in prior studies, and the objective of our research team, which is ultimately to incorporate it into medical devices, we have chosen a file format that imposes less burden on storage [51, 52]. Utilizing the HDF5 data format provides faster loading, increased storage efficiency, and compatibility with various programming languages [54, 55]. Throughout the study, we prioritized a compact model that occupied less storage space and enabled fast prediction of speech impairments. Employing MobileNetV3, a light-weighting and high-performance model, ensures the efficient execution of mobile devices [56]. We adapted an EfficientAT model [33, 34] as a reference, tailored to our specific data environment.

Lastly, the small volume of 3 cc was chosen for the protocol of this study in order to minimize the burden on patients during dysphagia assessments and to ease their consumption. The volume limitation of 3 cc was established based on our team's prior research, where kinematic analysis of VFSS images showed no significant difference in muscles (suprahyoid muscle, retrohyoid muscle, thyrohyoid muscle, sternothyroid muscle) activation duration, peak amplitude, and other parameters between 2 and 5 cc volumes [57]. Additionally, a systematic review related to dysphagia assessments indicated that many studies employed 3 cc [22]. These findings collectively informed the decision to set the volume at 3 cc.

This study developed a model to predict dysphagia—aspiration based on the postprandial voice. The expected benefits of this study are as follows. First, by determining the occurrence of aspiration and providing clinicians with more parameters through voice, it enhances the clinical utility compared to previous studies. Second, it is anticipated that the diagnosis time for both outpatient and inpatient cases will be significantly reduced, providing additional diagnostic parameters for a more accurate assessment of dysphagia. Third, this study is expected to lay the groundwork for designing diagnostic, treatment, and management systems by integrating them with future developments, such as a mobile application-based dysphagia meal guide monitoring system.

### Limitations

This study has several limitations. First, owing to the limited availability of voice data for individuals with dysphagia, we did not create a validation set, instead, we used a 9:1 training-to-testing data split (10-fold cross-validation). Second, due to the limited number of recruited female aspiration subjects, the female model showed lower performance compared with the combined model and male model. Third, voice data collection for healthy individuals and patients with dysphagia occurred in different environments and with varying numbers of participant, whereas the diet types were not standardized. Fourth, we addressed limitations in collecting clinical-normal data by recruiting general population participants, including those recorded with various devices and positions. Device bias was ruled out with cosine similarity consistently exceeding 0.8 after preprocessing. Fifth, this study aims to develop a voice-based disease prediction algorithm for integration into mobile and medical devices, targeting dysphagia monitoring and intervention. Creating a lightweight model and optimizing audio formats for input were essential steps. The use of diverse recording devices resulted in a variety of audio formats (wav, m4a, mp3), necessitating standardized preprocessing. From the start, all data was converted to mp3 at 64kbps for efficient training. Minimal data loss was observed, as analyzed by the Peak Signal-to-Noise Ratio (PSNR) in Additional file 3: Table S3. However, the potential for data loss represents a limitation of this study, underscoring the need for further investigation. Sixth, as a machine learning model trained on mel-spectrograms, we faced limitations in understanding which aspects of the model were crucial for dysphagia aspiration prediction. Consequently, we encountered a limitation in measuring feature importance, making it challenging to determine the significance of specific features in our model. In future studies, we aim to develop a more predictive model with better performance by recording a more diverse range of voices and diet types in patients with dysphagia, and comparing voice changes before and after meals.

## Conclusions

In this study, we utilized mel-spectrogram analysis of postprandial voice recordings and trained a MobileNetV3 model for mobile and medical device applications. This model showed high performance in predicting dysphagia aspiration, suggesting advancements in machine learning-based monitoring. Our study highlights the potential of voice analysis as valuable tool for screening, diagnosing, and monitoring dysphagia. It simplifies analysis compared to traditional methods like VFFS or FEES. Patients can also record their voices at home for self-monitoring, providing clinicians with valuable everyday data to track patients’ conditions. Identifying aspiration in daily life can improve patient quality of life and lead to non-invasive, safer interventions.

### Supplementary Information

**Additional file 1: Table S1.** Results of cosine similarity between measured positions and recording devices in the study.

**Additional file 2: Table S2.** Cosine similarity in the different locations.

**Additional file 3: Table S3.** The MSE and PSNR before and after mp3 (64kbps) conversion.

## Data Availability

All data in this study is available after de-identification upon request. The data that support the findings of this study are available from the first author, Jung-Min Kim (owljm@snu.ac.kr), upon reasonable request.

## Abbreviations

VFSS: Videofluoroscopic swallowing study
FEES: Fiberoptic endoscopic evaluation of swallowing
FOIS: Functional oral intake scale
V-VST: Volume-viscosity swallow test
RAP: Relative average perturbation
PPQ: Pitch period quotient
SHIM: Shimmer percent
APQ: Amplitude perturbation quotient
NHR: Noise to harmonic ratio
MVI: Maximal voice intensity
MPT: Maximum phonation time
PAS: Penetration aspiration scale
VDS: Videofluoroscopic dysphagia scale
ASHA-NOMS: American speech-language-hearing association national outcome measurement system swallowing scale
SVM: Support vector machine
GMM: Gaussian mixture model
EfficientAT model: Efficient Pre-Trained CNNs for Audio Pattern Recognition (MIT license)
IIT: Investigator initiated trial
STROBE: The strengthening the reporting of observational studies in epidemiology
FT3: Fluid thickening with level 3
LF: Liquid food
SBD: Semi-blended diet
SF: Small fluid
YP: Yoplait
MSE: Mean squared error
PSNR: Peak signal-to-noise ratio
PaSST: Efficient Training of Audio Transformers with Patchout (Apache-2.0 license)
HDF5: Hierarchical data format version 5
STFT: Short-time fourier transform
FFT: Fast fourier transforms
LPCM: Linear pulse code modulation
MLP: Multi-layer perceptron
AUC: Area under the ROC curve
mAP: Mean average precision
MFCCs: Mel frequency cepstral coefficients
CNNs: Convolutional neural networks

## Code availability

If you need information about the code, you can request access from the first authors, Jung-Min Kim (owljm@snu.ac.kr) or Min-Seop Kim (tjqtjq0516@gmail.com).
