# Supplementary material for: Prediction of dysphagia aspiration through machine learning-based analysis of patients’ postprandial voices
Source: J Neuroeng Rehabil. 2024 Mar 30;21:43. doi: 10.1186/s12984-024-01329-6 (PMC10981344; doi:10.1186/s12984-024-01329-6)
Supplement: Supplementary file 1 — Additional file 1: Table S1. Results of cosine similarity between measured positions and recording devices in the study. [file 12984_2024_1329_MOESM1_ESM.docx]

**Table S1. Results of cosine similarity between measured positions and recording devices in the study**

|  | | **Mobile Device Positions** | | |
| --- | --- | --- | --- | --- |
|  |  | **The upper sleeve**  **of the clothing** | **Table** | **In front of the mouth** |
| **1) Sony Recorder (the upper sleeve of the clothing) vs Mobile Devices** | | | | |
| **Sony Recorder**  **(the upper sleeve**  **of the clothing)** | **Samsung**  **Mobile Devices** | 0.8683 | 0.8770 | 0.8873 |
|  | **iPhone**  **Mobile Devices** | 0.9531 | 0.9618 | 0.9618 |
| **2) Samsung Mobile Devices vs iPhone Mobile Devices** | | | | |
|  | | **iPhone Mobile Devices** | | |
|  |  | **The upper sleeve**  **of the clothing** | **Table** | **In front of the mouth** |
| **Samsung**  **Mobile Devices** | **The upper sleeve**  **of the clothing** | 0.9491 | 0.8700 | 0.8482 |
|  | **Table** | 0.9464 | 0.8867 | 0.8709 |
|  | **In front of the mouth** | 0.9403 | 0.9061 | 0.9078 |

* Before conducting the research, we simultaneously recorded using mobile phone models (Samsung and iPhone) and a Sony recorder at three positions: the upper sleeve of the clothing, on a table, and in front of the mouth. Additionally, it’s important to note that the Sony recorder was exclusively used by the Videofluoroscopic Swallow Study (VFSS) examiner and was only measured at the position of the upper sleeve of the clothing. Therefore, data for the Sony recorder were exclusively utilized from this location.

** The recordings were then preprocessed in the same method as in our study, converted into Mel-spectrograms, and their cosine similarity was measured. The results, all above 0.8, confirmed that there was minimal bias due to the device and position used.

*** Cosine similarity assesses Mel spectrogram similarity by measuring the angular distance between vectors, reflecting how audio signal frequency patterns compare, calculated through the dot product of vectors normalized by their magnitudes.
