# Supplementary material for: Prediction of dysphagia aspiration through machine learning-based analysis of patients’ postprandial voices
Source: J Neuroeng Rehabil. 2024 Mar 30;21:43. doi: 10.1186/s12984-024-01329-6 (PMC10981344; doi:10.1186/s12984-024-01329-6)
Supplement: Supplementary file 2 — Additional file 2: Table S2. Cosine similarity in the different locations. [file 12984_2024_1329_MOESM2_ESM.docx]

**Table S2. Cosine similarity in the different locations**

|  | **The upper sleeve**  **of the clothing** | **Table** | **In front of the mouth** |
| --- | --- | --- | --- |
| **Sony Recorder** | | | |
| The upper sleeve of the clothing | 1.0000 | 0.9679 | 0.9484 |
| Table | 0.9679 | 1.0000 | 0.9583 |
| In front of the mouth | 0.9484 | 0.9583 | 1.0000 |
| **Samsung Mobile Device** | | | |
| The upper sleeve of the clothing | 1.0000 | 0.9898 | 0.9698 |
| Table | 0.9898 | 1.0000 | 0.9829 |
| In front of the mouth | 0.9698 | 0.9829 | 1.0000 |
| **iPhone Mobile Device** | | | |
| The upper sleeve of the clothing | 1.0000 | 0.9498 | 0.9322 |
| Table | 0.9498 | 1.0000 | 0.9719 |
| In front of the mouth | 0.9322 | 0.9719 | 1.0000 |

* This table, similar to Supplement Table 1, presents the impact of measurement positions based on cosine similarity among devices, using data converted into Mel-spectrograms as the preprocessing method utilized in this study. With similarity scores above 0.9, the results indicate that the impact of measurement positions within the same device is minimal.
