# Supplementary material for: Prediction of dysphagia aspiration through machine learning-based analysis of patients’ postprandial voices
Source: J Neuroeng Rehabil. 2024 Mar 30;21:43. doi: 10.1186/s12984-024-01329-6 (PMC10981344; doi:10.1186/s12984-024-01329-6)
Supplement: Supplementary file 3 — Additional file 3: Table S3. The MSE and PSNR before and after mp3 (64kbps) conversion. [file 12984_2024_1329_MOESM3_ESM.docx]

**Table S3. The MSE and PSNR before and after mp3 (64kbps) conversion**

| **Criteria** | **Audio File Format** | **Original Files** | **Converted to Mono Files (mp3, 64kbps)** | **MSE (Mean Squared Error)** ^*^ | | **PSNR (Peak Signal-to-Noise Ratio, dB)** ^*^ | |
| --- | --- | --- | --- | --- | --- | --- | --- |
|  |  |  |  | **Mean±SD** | **(95% CI)** | **Mean±SD** | **(95% CI)** |
| **Combined data (Male + Female)** | | | | | | | |
| Total  (Normal +Aspiration) | Total^**^ | 403 | 673 | 0.0002±0.0002 | (0.0002, 0.0002) | 35.95±3.76 | (35.67, 36.24) |
|  | wav | 259 | 518 | 0.0002±0.0003 | (0.0002, 0.0002) | 34.87±3.25 | (34.59, 35.15) |
|  | m4a | 136 | 147 | 0.0001±0.0001 | (0.0000, 0.0001) | 39.52±2.99 | (39.03, 40.00) |
|  | mp3 | 8 | 8 | 0.0001±0.0002 | (0.0000, 0.0002) | 40.40±4.47 | (36.67, 44.13) |
| Normal | Total^**^ | 210 | 287 | 0.0001±0.0003 | (0.0001, 0.0002) | 37.91±3.66 | (37.48, 38.33) |
|  | wav | 66 | 132 | 0.0002±0.0004 | (0.0001, 0.0003) | 35.97±3.33 | (35.39, 36.54) |
|  | m4a | 136 | 147 | 0.0001±0.0001 | (0.0000, 0.0001) | 39.52±2.99 | (39.03, 40.00) |
|  | mp3 | 8 | 8 | 0.0001±0.0002 | (0.0000, 0.0002) | 40.40±4.47 | (36.67, 44.13) |
| Aspiration | Total^**^ | 193 | 386 | 0.0002±0.0002 | (0.0002, 0.0002) | 34.50±3.14 | (34.19, 34.81) |
|  | wav | 193 | 386 | 0.0002±0.0002 | (0.0002, 0.0002) | 34.50±3.14 | (34.19, 34.81) |
|  | m4a | 0 | 0 | - | - | - | - |
|  | mp3 | 0 | 0 | - | - | - | - |
| **Male data** | | | | | | | |
| Total  (Normal +Aspiration) | Total^**^ | 388 | 388 | 0.0003±0.0003 | (0.0002, 0.0003) | 33.59±2.56 | (33.34, 33.85) |
|  | wav | 348 | 348 | 0.0003±0.0003 | (0.0003, 0.0003) | 33.22±2.30 | (32.97, 33.46) |
|  | m4a | 40 | 40 | 0.0001±0.0001 | (0.0001, 0.0002) | 36.86±2.32 | (36.12, 37.60) |
|  | mp3 | 0 | 0 | - | - | - | - |
| Normal | Total^**^ | 64 | 94 | 0.0003±0.0005 | (0.0002, 0.0004) | 34.57±3.09 | (33.94, 35.20) |
|  | wav | 27 | 54 | 0.0004±0.0006 | (0.0002, 0.0005) | 32.88±2.42 | (32.22, 33.54) |
|  | m4a | 37 | 40 | 0.0001±0.0001 | (0.0001, 0.0002) | 36.86±2.32 | (36.12, 37.60) |
|  | mp3 | 0 | 0 | - | - | - | - |
| Aspiration | Total^**^ | 147 | 294 | 0.0003±0.0002 | (0.0002, 0.0003) | 33.28±2.28 | (33.02, 33.54) |
|  | wav | 147 | 294 | 0.0003±0.0002 | (0.0002, 0.0003) | 33.28±2.28 | (33.02, 33.54) |
|  | m4a | 0 | 0 | - | - | - | - |
|  | mp3 | 0 | 0 | - | - | - | - |
| **Female data** | | | | | | | |
| Total  (Normal +Aspiration) | Total^**^ | 192 | 285 | 0.0001±0.0001 | (0.0000, 0.0001) | 39.17±2.58 | (38.87, 39.47) |
|  | wav | 85 | 170 | 0.0001±0.0001 | (0.0001, 0.0001) | 38.27±2.04 | (37.96, 38.57) |
|  | m4a | 99 | 107 | 0.0000±0.0000 | (0.0000, 0.0000) | 40.51±2.58 | (40.02, 41.00) |
|  | mp3 | 8 | 8 | 0.0001±0.0002 | (0.0000, 0.0002) | 40.40±4.47 | (36.67, 44.13) |
| Normal | Total^**^ | 146 | 193 | 0.0000±0.0001 | (0.0000, 0.0001) | 39.53±2.68 | (39.15, 39.91) |
|  | wav | 39 | 78 | 0.0001±0.0001 | (0.0001, 0.0001) | 38.11±1.87 | (37.68, 38.53) |
|  | m4a | 99 | 107 | 0.0000±0.0000 | (0.0000, 0.0000) | 40.51±2.58 | (40.02, 41.00) |
|  | mp3 | 8 | 8 | 0.0001±0.0002 | (0.0000, 0.0002) | 40.40±4.47 | (36.67, 44.13) |
| Aspiration | Total^**^ | 46 | 92 | 0.0001±0.0001 | (0.0001, 0.0001) | 38.40±2.17 | (37.95, 38.85) |
|  | wav | 46 | 92 | 0.0001±0.0001 | (0.0001, 0.0001) | 38.40±2.17 | (37.95, 38.85) |
|  | m4a | 0 | 0 | - | - | - | - |
|  | mp3 | 0 | 0 | - | - | - | - |

* We measured the degree of information loss (difference in data) between the original data and data compressed to mp3 at 64kbps in terms of Mean Squared Error (MSE) and Peak Signal-to-Noise Ratio (PSNR), as described above. Both MSE and PSNR are analytical methods used to compare the quality of voice compression. They are calculated using the following formulas respectively: MSE = mean((original data waveform - transformed data waveform (mp3, 64kbps))^2^), and PSNR = 20 X log_10_((maximum possible value of the original data signal) / √(MSE)). The MSE is a statistical measure without units, and the unit of PSNR is expressed in dB.

** In the Audio File Format, "Total" refers to the statistical data for all types of audio data (wav, m4a, mp3).

*** In the table, we compared the differences between the original data (note that since the original data is audio data, voices recorded in stereo were divided into two mono forms, adhering to the original data format) and the data used in this analysis in mp3, 64kbps (mono) format, from the perspectives of MSE (Mean Squared Error) and PSNR (Peak Signal-to-Noise Ratio). The differences in the data were examined through the disparities between the waveforms of the source audio itself, thereby assessing the degree of loss. The average loss rate for the entire dataset was found to be 0.0002±0.0002 by MSE and 35.95±3.76 by PSNR, indicating that the loss rate is not significant as the MSE is close to 0 and the PSNR is above 30dB.
